# Replication stress induced site-specific phosphorylation targets WRN to the ubiquitin-proteasome pathway

**DOI:** 10.18632/oncotarget.6659

**Published:** 2015-12-18

**Authors:** Fengtao Su, Souparno Bhattacharya, Salim Abdisalaam, Shibani Mukherjee, Hirohiko Yajima, Yanyong Yang, Ritu Mishra, Kalayarasan Srinivasan, Subroto Ghose, David J. Chen, Steven M. Yannone, Aroumougame Asaithamby

**Affiliations:** ^1^ Department of Radiation Oncology, University of Texas Southwestern Medical Center, Dallas, Texas, USA; ^2^ Department of Psychiatry, University of Texas Southwestern Medical Center, Dallas, Texas, USA; ^3^ Life Science Division, Lawrence Berkeley National Laboratory, Berkeley, California, USA; ^4^ Research Center for Charged Particle Therapy, National Institute of Radiological Sciences, Chiba, Japan

**Keywords:** Werner syndrome protein, Werner syndrome, replication stress, post-translational modification, chromosome instability, Gerotarget

## Abstract

Faithful and complete genome replication in human cells is essential for preventing the accumulation of cancer-promoting mutations. WRN, the protein defective in Werner syndrome, plays critical roles in preventing replication stress, chromosome instability, and tumorigenesis. Herein, we report that ATR-mediated WRN phosphorylation is needed for DNA replication and repair upon replication stress. A serine residue, S1141, in WRN is phosphorylated *in vivo* by the ATR kinase in response to replication stress. ATR-mediated WRN S1141 phosphorylation leads to ubiquitination of WRN, facilitating the reversible interaction of WRN with perturbed replication forks and subsequent degradation of WRN. The dynamic interaction between WRN and DNA is required for the suppression of new origin firing and Rad51-dependent double-stranded DNA break repair. Significantly, ATR-mediated WRN phosphorylation is critical for the suppression of chromosome breakage during replication stress. These findings reveal a unique role for WRN as a modulator of DNA repair, replication, and recombination, and link ATR-WRN signaling to the maintenance of genome stability.

## INTRODUCTION

Werner syndrome (WS) is a rare hereditary disease characterized by the premature onset of aging and a predisposition to a broad spectrum of rare cancers [1, 2]. Primary cells derived from WS patients exhibit elevated levels of chromosomal translocations, inversions, and large deletions with a high spontaneous mutation rate [3, 4]. Furthermore, WS cells are hypersensitive to several types of DNA damaging agents including 4-nitroquinoline-1-oxide, cross-linking agents (such as mitomycin C and cisplatin), camptothecin, and hydroxyurea [5-7]. Moreover, WS cells display a prolonged S-phase, impaired replication fork progression [8-10], and unstable replication forks [11]. These data indicate that the protein defective in WS, Werner syndrome protein (WRN), plays a role in genome stability maintenance pathways; however, the exact molecular contribution of WRN to the suppression of genomic instability is unclear.

WRN belongs to the RecQ DNA helicase family. WRN is unique among all RecQ helicases because of its N-terminal 3′ to 5′ exonuclease activity [12]. WRN exonuclease functions on a variety of structured DNA substrates, including bubbles, stem-loops, forks, and Holiday junctions. In addition, WRN exonuclease acts on RNA-DNA duplexes, suggesting a role for WRN in DNA replication, recombination, and repair [13]. The 3′ to 5′ DNA helicase activity of WRN [14] shows substrate specificity similar to exonuclease, suggesting that the two enzymatic activities have coordinated functions [15, 16] WRN forms dynamic sub-complexes with different factors involved in multiple biological processes. WRN physically interacts with Nijmegen breakage syndrome protein (NBS1) [17], MRE11 nuclease [18], Rad51 [19], RPA2 [20], XPG [21], NEIL1 [22], and PCNA [23]. In addition, WRN also interacts with ATR [19] and DNA-dependent protein kinase catalytic subunit (DNA-PKcs) [24, 25]. Importantly, mutations in many of these genes lead to disorders associated with cancer development.

WRN is post-translationally modified by phosphorylation, sumoylation, and acetylation in response to stress [26]. Post-translational modifications of WRN may affect its interaction with other protein partners, its stability, and its subcellular localization. WRN is phosphorylated by ATM, ATR [27, 28], and DNA-PKcs [24, 25, 29]. ATR/ATM-mediated WRN phosphorylation is required for check-point activation [30] and replication fork recovery [27] in response to replication stress. DNA-PKcs-dependent WRN phosphorylation is important for re-localization of WRN in the nucleoli and for its DNA repair and nuclease activities [24, 25, 29]. Additionally, WRN acetylation facilitates its translocation from the nucleoli to the nucleoplasm, and regulates its enzymatic activities [31] and its stability by inhibiting ubiquitination [32]. The impact of these modifications on WRN functions in response to replication stress is still unclear.

In this study we showed that serine 1141 of the WRN protein is phosphorylated by the ATR kinase in response to replication stress, using a combination of mass spectrometry and phospho-specific antibodies. ATR-mediated WRN phosphorylation leads to reversible interaction of WRN with replication-associated DNA double-stranded breaks (DSBs). This reversible DNA-WRN interaction facilitates replication fork processing, replication-associated DSB repair, and chromosome stability maintenance after replication stress. Importantly, ATR-mediated phosphorylation targets WRN for ubiquitination and degradation. Our findings reveal that ATR-mediated WRN phosphorylation is required for DNA replication, repair, and recombination, and ultimately for maintenance of genome stability during replication stress.

## RESULTS

### The Werner syndrome protein is phosphorylated at serine 1141 by ATR in response to replication-associated DNA double-strand breaks

WRN is phosphorylated by DNA-PKcs [24, 25, 29], ATM, and ATR [27] both *in vitro* and *in vivo*. To understand the biological significance of WRN phosphorylation in genome stability maintenance, we first sought to identify the residues that are phosphorylated in WRN using mass spectrometry (MS). Highly purified WRN [24] previously dephosphorylated during purification was incubated with DNA-PKcs; this reaction was expected to phosphorylate all serines and threonines on the protein. This phosphorylated WRN was then isolated from SDS-PAGE gels and subjected to MS proteomic analysis to identify modified sites [33]. Using this approach, we identified several amino acid residues in WRN that were phosphorylated by DNA-PKcs *in vitro*. The serine at position 1141 was unambiguously identified as a phosphorylation site. Subsequently, we generated rabbit polyclonal antibodies to a 13-residue peptide that contained a phospho-serine at the 1141 position. The serum containing phospho-specific antibodies was purified using immobilized non-phosphorylated and phosphorylated forms of the antigenic peptide.

**Figure 1:**
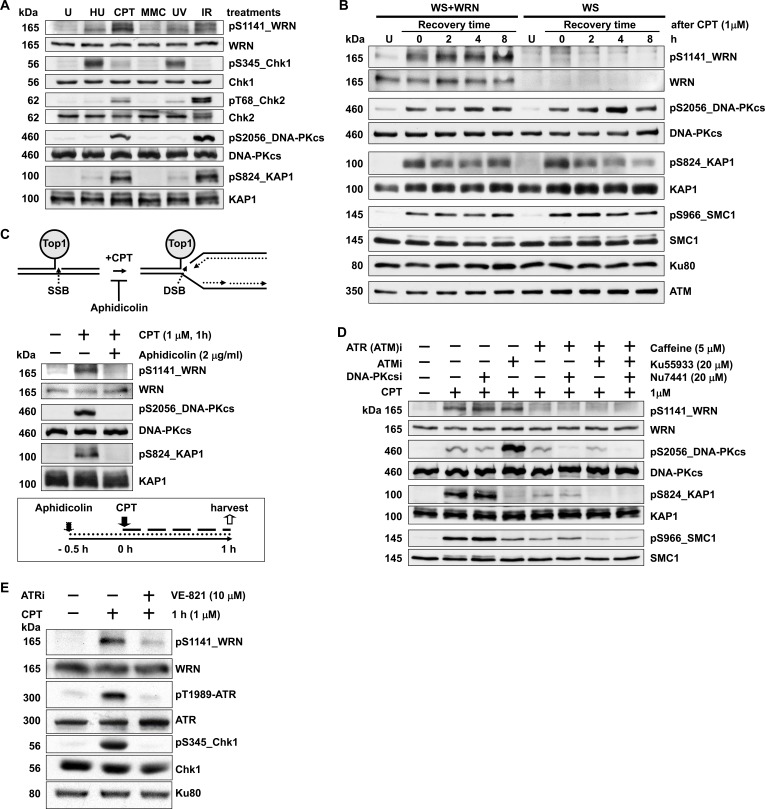
WRN is phosphorylated at serine 1141 by ATR in response to replication-associated DSBs **A.** WRN is heavily phosphorylated at S1141 in response to CPT treatment of cells. HeLa cells were mock-treated or treated with 3 mM HU, 5 μM MMC, 10 J/m^2^ ultraviolet light (UV), or 5 Gy ionizing radiation (IR). Cells were harvested 1 h after treatment and analyzed for WRN, Chk1, Chk2, DNA-PKcs and KAP1 phosphorylation events by western blotting. **B.** WRN is phosphorylated at S1141 in response to replication stress. hTERT immortalized WS cells stably transfected with empty vector (WS) and WS cells stably expressing wild-type WRN (WS+WT) were mock-treated (lane U) or treated with 1 μM CPT for 1 h. Cells were harvested at indicated times and analyzed for CPT-induced phosphorylation of WRN at S1141 using anti-pS1141 antibodies by western blotting. Western blots for total and phosphorylated DNA-PKcs, KAP1, SMC1, and Chk2 were also performed. **C.** Phosphorylation of WRN at S1141 is triggered by replication-associated DSBs. HeLa cells were pre-treated with aphidicolin (2 μg/ml) for 0.5 h and then either mock- or CPT-treated (1 μM) for 1 additional h. Cells were harvested after CPT treatment, and phosphorylation of WRN, DNA-PKcs, and KAP1 was analyzed by western blotting. A graphical sketch of CPT-induced DNA double-strand breaks and experimental design are shown above and below images of western blots, respectively. **D.** WRN phosphorylation at S1141 in response to replication-associated DSBs is sensitive to caffeine treatment. HeLa cells were pre-treated with 5 μM caffeine, 20 μM Ku55933, or 20 μM Nu7441 either alone or in combination for 1 h and then mock- or CPT-treated (1 μM) for 1 h. Cells were harvested 1 h after treatment and analyzed for phosphorylation of WRN, DNA-PKcs, KAP1, and SMC1 by western blotting. **E.** Phosphorylation of WRN at S1141 in response to replication-associated DSBs is mediated by ATR. HeLa cells were treated with either DMSO or 10 μM ATR (VE-821) -specific inhibitor. Two h after exposure to ATR inhibitor, cells were either mock- or CPT-treated (1 μM) for 1 h. The cells were harvested 1 h after the treatment and examined for phosphorylation of WRN, ATR and Chk1 by western blotting.

To investigate the specific type of DNA damage that induces phosphorylation of WRN in cells, we exposed HeLa cells to the replication stress inducers camptothecin (CPT) and hydroxyurea (HU), the interstrand cross-linking agent mitomycin C (MMC), ultraviolet light (UV) to generate cyclobutane-pyrimidine dimers, and ionizing radiation (IR) to induce DSBs. The most intense pS1141 signal was observed in cells treated with CPT; markedly weaker signals were observed in cells treated with HU, MMC, and UV; the pS1141 signal in IR-treated cells was intermediate between mock- and CPT-treated cells (Figure 1A). DNA-PKcs (S2056) and ATM substrates (KAP1 and Chk2) showed the same phosphorylation patterns across this panel of treatments.

To determine the specificity of the affinity-purified anti-pS1141 antibodies, hTERT immortalized WS cells and WS cells complemented with wild-type WRN (WS+WT) were treated with 1 μM CPT for 1 h and harvested at different times after treatment. As shown in Figure 1B, the pS1141 signal was readily detectable in CPT-treated WS+WT cells, but not in CPT-treated WS cells. Furthermore, phosphorylation of DNA-PKcs, ATM, and ATR targets was not affected in CPT-treated WS cells (Figure 1B), suggesting that the absence of pS1141 signal in WS cells was not due to defective DNA-PK, ATM, or ATR activation. Thus, anti-pS1141 antibodies specifically recognize WRN phosphorylated at S1141.

CPT treatment results in the formation of DSBs when DNA replication forks collide with the Top1 cleavage complex trapped by CPT [34, 35]. Treatment of cells with DNA polymerase inhibitor aphidicolin inhibits the formation of replication-mediated DSBs [34, 35] (Figure 1C, top). Therefore, we treated cells with 2 μg/ml aphidicolin to test whether CPT-induced WRN phosphorylation at S1141 is due to formation of replication-mediated DSBs (Figure 1C, bottom). Pre-treatment of HeLa cells with aphidicolin blocked CPT-induced WRN phosphorylation at S1141 and similarly reduced the phosphorylation of DNA-PKcs and KAP1 (Figure 1C, center). Thus, WRN is phosphorylated at S1141 in response to replication-associated DSBs.

To identify the kinase or kinases responsible for WRN phosphorylation at S1141, we pre-treated HeLa cells with caffeine, which inhibits ATM and ATR [36]; with Ku55933, which inhibits ATM [37]; or with Nu7441, which inhibits DNA-PKcs [38]. Pre-treatment of HeLa cells with the DNA-PKcs inhibitor (Nu7441) or the ATM inhibitor (Ku55933) did not significantly reduce the pS1141 signal in response to CPT treatment (Figure 1D). In contrast, pre-treatment of cells with caffeine at a dose of 5 μM inhibited both ATM and ATR and significantly reduced the CPT-induced pS1141 signal (Figure 1D, compare lanes 2 and 5). Caffeine inhibits both ATR and ATM kinase activities and pre-treatment of cells only with the ATM inhibitor (Ku55933) did not significantly reduce pS1141 signal, suggesting that ATR phosphorylates WRN at S1141. To confirm these results, we inhibited kinase activity of ATR in HeLa cells using an ATR-specific small molecule inhibitor (VE-821) and then analyzed S1141 phosphorylation. The pS1141 signal was markedly reduced in CPT-treated ATR-inhibited cells relative to DMSO-treated control cells (Figure 1E). Thus, these results strongly suggest that ATR is the major kinase that phosphorylates WRN at S1141 in response to replication-associated DSBs.

### WRN phosphorylation at S1141 facilitates its reversible interaction with replication-associated DSBs

**Figure 2:**
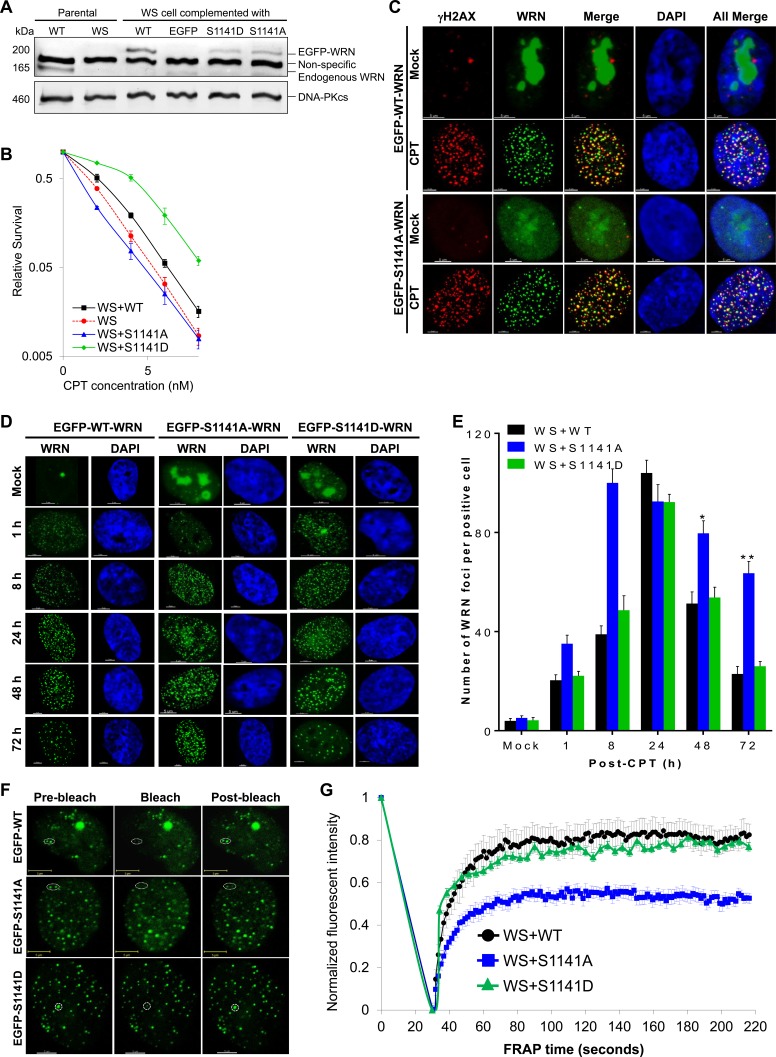
ATR-mediated WRN phosphorylation facilitates reversible interaction of WRN with replication-associated DSBs **A.** Expression of Flag-EGFP tagged WT, S1141A, and S1141D WRN in WS cells. WS cells were infected with retrovirus carrying Flag-EGFP-tagged WT, S1141A, or S1141D WRN. Cells were stably selected with puromycin. Stable clones were examined for Flag/EGFP WRN expression using anti-Flag and anti-WRN antibodies by western blotting. DNA-PKcs was used as a loading control. **B.** S1141 phosphorylation is important for CPT sensitivity, but not serine itself. WS, WS+WT, WS+S1141A, and WS+S1141D cells were treated with different concentrations of CPT for 72 h and where analyzed by colony formation assay. Each data point in the graph is the average of three independent experiments. Error bars represent STDEV. **C.** WRN is recruited to the sites of replication-associated DSBs independently of its S1141 phosphorylation. WS cells stably expressing EGFP-tagged WT and S1141A WRN were treated with 1 μM CPT for 1 h. After 8 h, cells were fixed with 4% paraformaldehyde and incubated with anti-γH2AX antibodies for indirect immunostaining. Representative three-dimensional deconvoluted confocal images are shown. Scale bars are 5 μm. **D.**-**E.** WRN phosphorylation regulates dissociation properties of WRN from the sites of replication-associated DSBs. **D.** Representative high-resolution confocal images show WT, S1141A, and S1141D WRN foci in WS cells. **E.** Numbers of WT, S1141A, and S1141D WRN foci at indicated times after CPT-treatment. WS cells stably expressing EGFP-tagged WT or S1141A or S1141D WRN were treated with 1 μM CPT for 1 h. Cells were fixed with 4% paraformaldehyde at indicated times and observed with confocal microscopy. The number of EGFP-WRN foci in each cell was determined using the spot-detection function of the Imaris Software. The average number of foci in 50 cells in each sample and time points from three independent experiments were used for the calculation. Error bars represent STDEV. * indicates P<0.01; ** indicate P<0.001. Scale bars are 5 μm. **F.**-**G.** ATR-mediated WRN phosphorylation modulates the association of WRN with replication-associated DSBs. **F.** Representative confocal images show WT, S1141A and S1141D WRN foci before and after photobleaching (representative foci are circled). **G.** FRAP curves for EGFP-WT, S1141A, and S1141D WRN focus/foci. WS cells stably expressing EGFP-tagged WT, S1141A, or S1141D WRN were treated with 1 μM CPT for 1 h. Cells were allowed to recover for 24 h. One or two randomly selected WRN focus/foci were photobleached and the recovery of fluorescence signal was captured using a live cell confocal microscope. In every image, average fluorescent intensities of the photobleached EGFP-WRN foci were measured as a function of time and then divided by the average fluorescent intensity measured elsewhere in the cell as a function of time. The normalized FRAP curves for each cell were obtained by dividing the EGFP-WRN fluorescent intensities in each spot after the photobleaching by the pre-bleach intensity; the pre-bleach intensity was set to one. Each data point depicted in the graph is the average of 30 independent normalized measurements. The error bars represent SEM.

To identify the biological functions of WRN phosphorylation at S1141, we abolished this serine phosphorylation site in full-length WRN by replacing the serine codon at 1141 with alanine (S1141A). To demonstrate that the phenotypes associated with the S1141A mutation were due to the loss of serine phosphorylation rather than the disruption of the serine residue itself, we created phosphomimetic WRN by replacing the serine codon at 1141 with aspartic acid (S1141D). Subsequently, we created stable WS+S1141A and WS+S1141D cell lines. As shown in Figure 2A, WT, S1141A, and S1141D WRN proteins were expressed at similar levels in WS cells. Next, we verified whether the S1141A mutation abolished CPT-induced WRN S1141 phosphorylation using anti-phospho-S1141 antibodies. As expected, exogenous WT-WRN was phosphorylated at S1141 in response to CPT, but S1141A was not phosphorylated in response to CPT (Figure S1A). These results suggest that S1141A mutation completely abolished CPT-induced WRN phosphorylation at S1141.

Since WRN is phosphorylated at S1141 in response to CPT, we next tested whether the mutation in the WRN S1141 phosphorylation site renders cells sensitive to CPT. Similar to previous studies [5, 11], WS cells were more sensitive to CPT-induced cytotoxicity than the WS+WT cells (Figure 2B). Significantly, WS+S1141A cells were also sensitive to CPT. In contrast, the WS+S1141D cells, which express phosphomimetic WRN, were less sensitive to CPT-induced cytotoxicity than WS+WT cells (Figure 2B). Thus, WRN phosphorylation at S1141 is critical for cellular sensitivity to CPT exposure and that the increased sensitivity of WS+S1141A cells to CPT is due to lack of phosphorylation at position 1141 rather than to the serine itself.

Since WRN is phosphorylated on S1141 in response to replication-associated DSBs, we sought to determine whether WRN phosphorylation influences its recruitment to the sites of these DNA breaks. As shown in Figure 2C, both WT and S1141A WRN formed distinct foci after CPT treatment of WS cells stably expressing WT and S1141A-WRN, respectively. In addition, a majority of WT and S1141A WRN foci clearly overlapped with γH2AX foci in CPT-treated cells but not in mock-treated cells (Figure 2C). Thus, WRN is recruited to the sites of replication-associated DSBs independently of its S1141 phosphorylation status.

Subsequently, we investigated whether WRN S1141 phosphorylation affects its association or dissociation properties at replication-associated DSBs. We first evaluated WT, S1141A, and S1141D WRN foci kinetics in response to replication stress (Figure 2D). Quantification of WRN foci at the single-cell level revealed that the number of WT WRN foci gradually increased as a function of time, reaching a peak (104.02+5.16 foci per cell) at 24 h after CPT-treatment and then decreasing to ~30% of the maximum number after 72 h (Figure 2E). Similarly, the number of S1141D-WRN foci gradually increased as a function of time, reaching a maximum (92.25+3.16 foci per cell) at 24 h after CPT treatment and then decreased to 28% of the maximum number after 72 h (Figure 2E). In marked contrast, the number of S1141A WRN foci more rapidly reached a maximum (100.25+5.65 foci per cell) between 8 and 24 h after CPT treatment and did not decline; 63% of the maximum number of S1141A WRN foci persisted at 72 h after CPT exposure (Figure 2E). Thus, WRN phosphorylation at S1141 by ATR modulates its dissociation from DSBs during replication stress.

To corroborate these findings, we examined the stability of protein-DNA complexes formed by WT, S1141A, and S1141D WRN at replication-associated DSBs by monitoring fluorescence redistribution after photobleaching (FRAP) as described previously [39]. FRAP measurements revealed that EGFP-tagged WT WRN fluorescence at the sites of replication-associated DSB recovered very quickly, reaching ~75% of pre-bleach fluorescence intensity within 60 seconds (Figure 2F-2G). The rapid fluorescence recovery indicates a dynamic exchange between free WRN and WRN bound to DNA. The recovery rate of the S1141D-mutant WRN was nearly identical to that of WT WRN (Figure 2G). In contrast, EGFP-S1141A WRN fluorescence at the damaged sites recovered only ~42% of pre-bleach fluorescence intensity within 60 seconds (Figure 2G), indicating a markedly reduced rate of exchange between free WRN S1141A and that bound to replication-associated DSBs relative to WT WRN. Differences in recovery rates between WT/S1141D and S1141A WRN suggest that S1141 phosphorylation modulates reversible interaction of WRN with replication-associated DSBs. To further confirm the involvement of ATR mediated WRN phosphorylation in modulating the interaction of WRN with replication-associated DSBs, we pre-treated WS cells stably expressing EGFP-WT WRN with the ATR inhibitor (VE-821) and examined WRN dynamics by FRAP. Similar to S1141A WRN, we noticed that the WT-WRN fluorescence recovered only ~44% of pre-bleach fluorescence within 60 seconds (Figures S1B-S1C). Overall, these results support the idea that ATR-mediated WRN phosphorylation regulates interaction of WRN with replication-associated DSBs.

### ATR-mediated WRN phosphorylation facilitates Rad51-mediated homologous recombination repair of replication-associated DSBs

**Figure 3:**
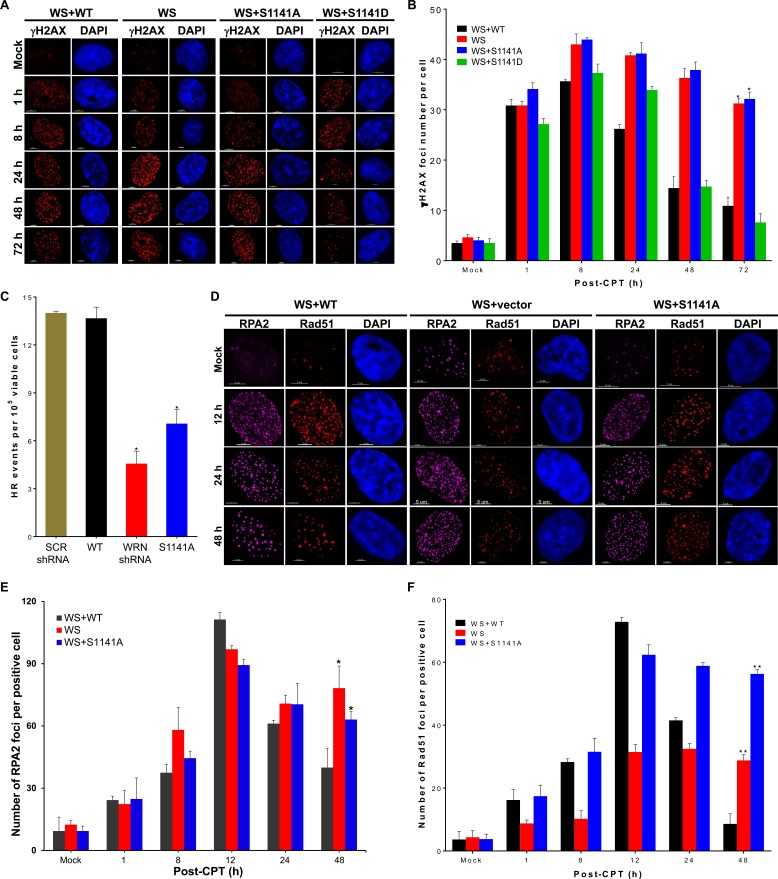
ATR-mediated WRN phosphorylation facilitates homologous recombination-mediated repair of replication-associated DSBs **A.**-**B.** WRN S1141 phosphorylation is important for the repair of replication-associated DSBs. **A.** Representative confocal images show γH2AX foci at indicated times after CPT-treatment. **B.** Graph shows the number of γH2AX foci at indicated times after CPT-treatment. WS, WS+WT, WS+S1141A, and WS+S1141D cells were treated with 1 μM CPT for 1 h. Cells were fixed with 4% paraformaldehyde at indicated times after the treatment and then immunostained with anti-γH2AX antibodies. The γH2AX foci in each cell were quantified using the spot-detection function of the Bitplane Imaris software. The average number of foci in 100 cells in each sample and at each time point from three independent experiments was used for the calculation. Error bars represent STDEV; * indicates P<0.01. Scale bars are 5 μm. **C.** WRN phosphorylation is important for homologous recombination (HR) repair. HT1080-1885 cells harboring a single copy of the I-SceI-inducible HR substrate were transfected with either I-SceI expression vector pCMV (3 × NLS) alone or with *WRN* shRNA, Flag tagged WT WRN, or S1141A WRN using Amaxa Nucleofector (Solution T, Program L005). Twenty four h after transfection, cells were allowed to form colonies in the presence of puromycin (1 μg/ml), and the HR frequency was calculated by measuring the number of colonies. Each data point in the graph is the average of three independent experiments. Error bars represent STDEV; * indicates *P* < 0.01. **D.**-**F.** WRN phosphorylation influences RPA2 and Rad51 foci dynamics at replication-associated DSBs. **D.** Representative confocal images show RPA2 and Rad51 foci at indicated times after CPT-treatment. **E.** The number of RPA2 foci at indicated times after CPT-treatment is shown. **F.** The number of Rad51 foci at indicated times after CPT-treatment is shown. WS, WS+WT, and WS+S1141A cells were treated with 1 μM CPT for 1 h. Cells were fixed with 4% paraformaldehyde at indicated times and then subjected to indirect immunostaining with anti-RPA2 or anti-RAD51 antibodies. High-resolution three-dimensional deconvoluted confocal images were used to quantify the number of RPA2 and Rad51 foci in each cell using the spot-detection function of the Imaris Software. The average number of foci in > 100 cells in each sample at each time point from three independent experiments were used for the calculation. Error bars represent STDEV; * indicates *P* < 0.01; ** indicate *P* < 0.001.

The reversible interaction of WRN with replication-associated DSBs may be necessary for the timely repair of replication-associated DSBs. To verify this idea, we investigated the repair of replication-associated DSBs in WS, WS+WT, WS+S1141A, and WS+S1141D cells after replication stress using γH2AX as a surrogate marker [40]. In CPT-treated WS+WT cells, the number of γH2AX foci per cell increased gradually and reached a peak around 8 h; the number of foci was reduced to 30.6% of this maximum after 72 h (Figures 3A-3B). Similarly, in CPT-treated WS+S1141D cells, the number of γH2AX foci per cell increased gradually, reaching a maximum around 8 h and reducing to 20.4% after 72 h (Figures 3A-3B). Dissolution kinetics of γH2AX foci in both WS and WS+S1141A cells differed from that of WS+WT and WS+S1141D cells (Figure 3B). Even 72 h after recovery from exposure to CPT, WS and WS+S1141A cells still had more than 70% of their maximum γH2AX foci number compared to WS+WT and WS+S1141D cells (Figure 3B). We further confirmed the involvement of WRN S1141 phosphorylation in replication-associated DSBs repair by a comet assay [41]. Similar to γH2AX foci resolution kinetics, the comet tail movement was longer in CPT-treated WS+S1141A cells relative to CPT-treated WS+WT cells (Figures S2A-S2B). Thus, these results imply that the reversible interaction of WRN with DNA is necessary for the repair of replication-associated DSBs.

As the homologous recombination (HR) pathway is the major active repair mechanism during replication [42], we tested whether ATR-mediated WRN phosphorylation is involved in HR-mediated DSB repair using an HR-reporter assay as previously described (Figures S2C-S2D) [43]. As shown in Figure 3C, shRNA-mediated depletion of WRN significantly reduced the number of HR events relative to control shRNA-transfected cells. Similarly, expression of S1141A WRN significantly decreased the number of HR frequencies as compared to expression of WT WRN (Figure 3C). Thus, ATR-mediated WRN phosphorylation functions in HR-mediated DSB repair.

HR is initiated by DNA-end resection followed by Rad51-dependent strand invasion into duplex DNA and the pairing of homologous DNA strands. Therefore, reduced levels of HR repair in WS+S1141A cells could be either due to defective DNA end-resection or inefficient recruitment of Rad51. We first examined DNA-end resection using RPA2 as a surrogate marker in WS, WS+WT, and WS+S1141A cells following replication stress (Figure 3D). In CPT-treated WS+WT cells, the number of RPA2 foci increased gradually and reached a maximum around 12 h (110.44+1.2 foci per cell) and then declined to 40+7.4 foci at 48 h (Figure 3E). In contrast, in CPT-treated WS and WS+S1141A cells the number of RPA2 foci rose more rapidly and reached a maximum around 12 h (95.55+2 and 91.21+2.8 foci per cell, respectively). In both WS and WS+S1141A cells, RPA2 foci persisted and were maintained near maximal levels (78.31+10.6 and 63.1+3.9 foci per WS and WS+S1141A cell, respectively) at 48 h (Figure 3E). Thus, the extent of DNA-end resection is independent of WRN-phosphorylation and the presence of persistent RPA2 foci both in WS and WS+S1141A cells reflects unrepaired replication-associated DSBs in these cells.

Subsequently, we examined Rad51 foci kinetics in WS, WS+WT, and WS+S1141A cells (Figures 3D and 3F). Like RPA2 foci, the number of RAD51 foci per cell increased gradually reaching a maximum around 12 h (72.9+1.4 foci per cell), and the levels were reduced to 11.8% of the maximum after 48 h in CPT-treated WS+WT cells (Figure 3F). Interestingly, Rad51 foci number did not decline to CPT-treated WS+WT levels in CPT-treated WS+S1141A cells (Figure 3F). Even after 48 h of recovery from exposure to CPT, WS+S1141A cells retained more than 90.3% of the Rad51 foci (Figure 3F). Furthermore, the presence of high-levels of Rad51 foci in WS+S1141A cells correlated well with the extent of persistent γH2AX and RPA2 foci in these cells. On the other hand, in agreement with data reported in a previous study [11], the number of Rad51 foci in CPT-treated WS cells was significantly lower than the number in CPT-treated WS+WT WS+S1141A WRN cells (Figure 3F). Thus, the presence of persistent Rad51 foci with reduced HR frequencies in S1141A cells reflect incomplete HR repair. We hypothesize that the reversible interaction of phosphorylated WRN with replication-associated DSBs facilitates Rad51-mediated HR repair.

### WRN phosphorylation at S1141 is critical for replication fork maintenance in response to replication stress

**Figure 4:**
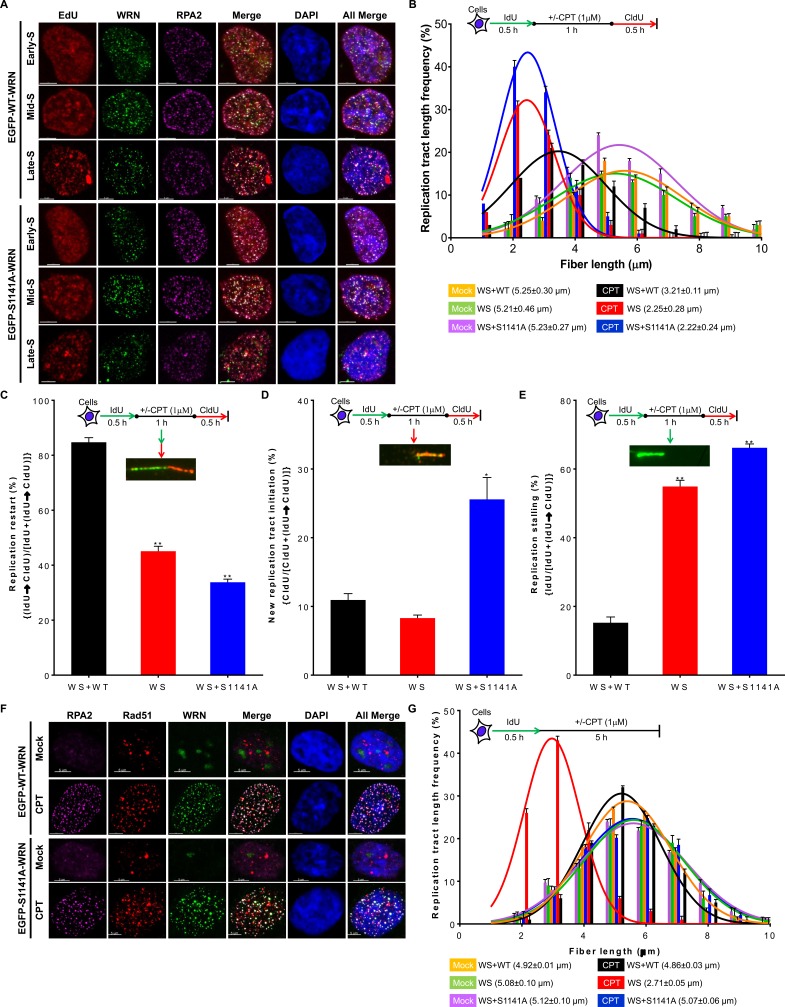
ATR-mediated WRN phosphorylation is critical for replication fork processes upon replication stress **A.** WRN is recruited to replication forks sites independently of its S1141 phosphorylation. WS cells stably expressing EGFP-tagged WT or EGFP-tagged S1141A WRN were pulse-labeled with 50 μM EdU for 90 min and then treated with 1 μM CPT for 1 h. After 8 h, cells were fixed with 4% paraformaldehyde and immunostained with anti-RPA2 antibodies. Subsequently, the Click-IT reaction was carried out to detect EdU signal. Representative three-dimensional deconvoluted confocal images are shown. Scale bars are 5 μm. **B.** Replication fork progression is inefficient in WS+S1141A cells in response to replication stress. DNA fiber length distributions in WS+WT, WS, and WS+S1141A cells are illustrated before and after CPT treatment. Cells were labeled with IdU for 30 min, treated with ± CPT (1 μM) for 60 min, and labeled with CIdU for another 30 min. DNA fibers were immunostained with anti-BrdU (rat and mouse) antibodies, images were captured using fluorescence microscopy, and IdU (mock-treated) and CldU (CPT-treated) tract lengths were measured using Axiovison Software. More than 200 DNA fibers were evaluated in each sample. Each data point is the average of three independent experiments. Error bars represent STDEV. **C.**-**E.** WRN phosphorylation suppresses new origin firing and replication fork stalling in response to replication stress. **C.** Percentages of replication fork restarts in CPT-treated WS+WT, WS, and WS+S1141A cells relative to mock-treated cells are shown; levels were evaluated using the following formula: (IdU→CldU)/[IdU+(IdU→CldU)]. **D.** The graph shows fold changes in new origin firing in CPT-treated WS+WT, WS, and WS+S1141A cells relative to mock-treated cells calculated using the formula: CldU/[CldU+(IdU→CldU)]. **E.** The graph shows fold changes in replication forks stalling in CPT-treated WS+WT, WS, and WS+S1141A cells relative to mock-treated cells evaluated using the formula: IdU/[IdU+(IdU→CldU)]. More than 100 DNA fibers in each sample were evaluated. Each data point is the average of three independent experiments. Error bars represent STDEV. * indicates *P* < 0.05; ** indicate *p* < 0.001. **F.** WRN co-localizes with RPA2 and RAD51 independently of S1141 phosphorylation in response to CPT-induced replication stress. WS cells stably expressing EGFP-tagged WT or EGFP-tagged S1141A WRN were treated with 1 μM CPT for 1 h. After 8 h, cells were fixed with 4% paraformaldehyde and incubated with anti-RPA2 and anti-RAD51 antibodies for indirect immunostaining. Representative confocal images are shown. Scale bars are 5 μm. **G.** Nascent DNA-strands are stable in WS+S1141A cells. Replicating DNA in WS, WS+WT, and WS+S1141A cells was labeled by incorporating IdU. Cells were exposed to 1 μM CPT for 1 h. The length of IdU tracts was measured 5 h after CPT-treatment. Frequency distributions of lengths over 100 DNA fibers were calculated in each group from three independent experiments. Error bars represent STDEV.

In addition to DSB repair, WRN also functions in replication fork processes [11]. Therefore, we examined whether WRN phosphorylation influences its recruitment to sites of perturbed replication forks. Similarly to a previous finding [11], WT and S1141A WRN clearly juxtaposed with the EdU-labeled replication sites in CPT-treated early-, mid-, and late-S-phase cells (Figure 4A). We confirmed these results using PCNA as a surrogate marker for replication forks. We found that WRN clearly juxtaposed with PCNA foci in CPT treated WS+EGFP-WT WRN cells (Figure S3). Additionally, both WT and S1141A WRN foci clearly co-localized with RPA2 in all S-phase cells (Figure 4A). Thus, recruitment of WRN to the sites of perturbed replication forks is independent of its phosphorylation at S1141. Therefore, it is reasonable to assume that the replication fork processes would be similar between WS+WT and WS+S1141A cells after replication stress. We monitored replication fork progression, stalling, and new origin firing and stability using a single-molecular DNA fiber technique [11]. As shown in Figure 4B, IdU tract lengths in mock-treated WS, WS+WT, and WS+S1141 cells were nearly the same (5.25+0.3, 5.21+0.46, and 5.23+0.27 μm, respectively). In contrast, CPT treatment reduced the CldU tract lengths relative to the IdU tract lengths in all tested cells, reflecting the slowing of replication fork elongation upon CPT treatment (Figure 4B). Notably, the DNA fiber lengths (CldU lengths) were significantly shorter in CPT-treated WS and WS+S1141A cells (2.25+0.28 and 2.22+0.24 μm in WS and WS+S1141A cells, respectively; *p* = 0.027 and *p* = 0.038) than in CPT-treated WS+WT cells (3.21+0.11 μm, Figure 4B). Furthermore, cell cycle analysis clearly showed that S1141 phosphorylation plays a critical role in the progression of S-phase cells upon collapsed replication forks (Figure S4). Thus, as shown previously [27], ATR-mediated WRN phosphorylation is involved in the recovery of replication forks in response to replication stress.

Subsequently, we evaluated the extent of the replication fork restart, new origin firing and stalling in CPT-treated cells by the sequential labeling of replicating DNA with IdU and CldU before and after CPT treatment, respectively. As shown in Figure 4C, 45.09+1.86% and 33.81+1.11% of all DNA fibers had both IdU and CldU tracts in CPT-exposed WS and WS+S1141A cells, respectively. In contrast, 84.74+1.67% fibers contained both IdU and CldU in CPT-treated WS+WT cells (Figure 4C). These results indicate that a greater proportion of replication forks fail to restart in CPT-treated WS and WS+S1141A cells as compared with CPT-exposed WS+WT cells. Intriguingly, we observed significantly elevated levels of DNA fibers containing only CldU tracts, representing new origins of replication, in WS+S1141A (25.58+3.18%, *p* = 0.0169) as compared with WS (8.31+0.44%) cells (Figure 4D). Thus, similar to a recent report analyzing Fanconi anemia complementation group I (FANCI) [44], ATR-mediated WRN phosphorylation is somehow involved in the suppression of dormant origin firing upon replication stress. Furthermore, a significantly higher percentage of DNA fibers contained only IdU tracts, representing stalled forks, in CPT-treated WS and WS+S1141A cells as compared with CPT-treated WS+WT (15.26+1.66%, 54.91+1.82%, and 66.19+1.11%, WS+WT, WS and WS+S1141A cells, respectively, *p* = 0.002 and 0.0005, Figure 4E). Thus, a greater proportion of replication forks break in CPT-treated WS and WS+S1141A cells than in CPT-treated WS+WT cells. Taken together, these results suggest that S1141 phosphorylation is critical for replication fork restart and for the suppression of both new origin firing and replication fork collapse in response to replication stress.

Evidence shows that WRN functions with Rad51 to protect nascent DNA strands in response to replication stress [11]. Furthermore, stable association of Rad51 with replication-associated DSBs stabilizes nascent DNA strands in the absence of WRN. Therefore, it is possible that the persistent binding of Rad51 with replication-associated DSBs prevents shortening of nascent DNA strands in WS+S1141A cells but does not in WS cells. To validate this notion, we first sought to determine whether WRN phosphorylation influences its co-localization with Rad51. As shown in Figure 4F, there was a clear co-localization of WT and S1141A WRN foci with Rad51 foci in CPT-treated cells, implying that WRN phosphorylation at S1141 is not required for its co-localization with Rad51. Subsequently, we measured nascent DNA tract lengths in WS, WS+WT, and WS+S1141A cells. As reported previously [11], nascent DNA strands were significantly shorter in CPT-treated WS cells than in CPT-treated WS+WT cells (2.71+0.05 μm and 4.86+0.03 μm, respectively, *p* = 0.0010, Figure 4G). In contrast, nascent DNA tract lengths in CPT-treated WS+S1141A cells were similar to those in CPT-treated WS+WT cells (5.07+0.06 μm and 4.86+0.03 μm, respectively, p < 0.062, Figure 4G). Thus, reversible interaction of phosphorylated WRN with replication-associated DSBs facilitates proper replication fork processes following replication stress.

### ATR-dependent WRN phosphorylation facilitates WRN ubiquitination

**Figure 5:**
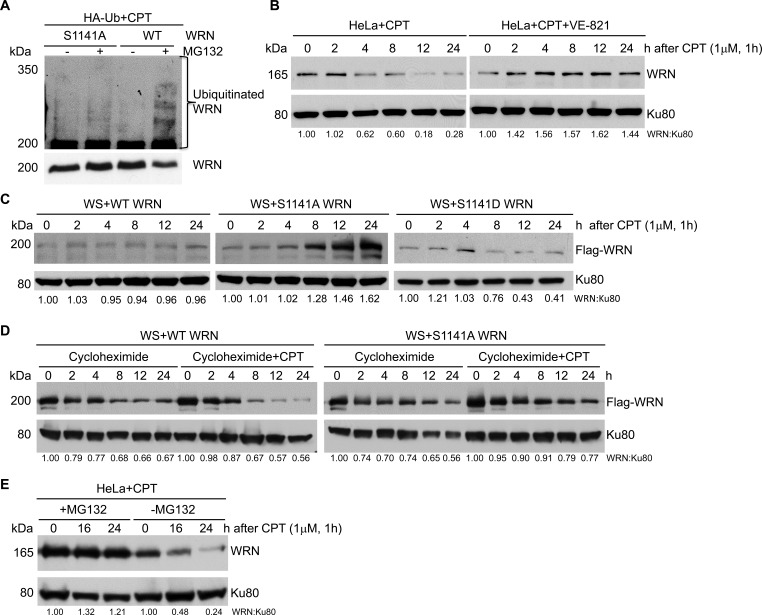
ATR-mediated WRN S1141 phosphorylation leads to ubiquitination of WRN and subsequent degradation after replication stress **A.** WRN is ubiquitinated in response to replication stress. Exponentially growing HeLa cells were transfected with HA-tagged ubiquitin. After 36 h cells were co-treated with 25 μM MG132 and 1 μM CPT for 1 h, washed with 1x PBS, and maintained in growth medium containing MG132 for an additional 16 h. The total cell extract was subjected to immunoprecipitation using anti-flag antibodies and the immune complex was probed with anti-WRN and anti-HA antibodies. **B.** WRN is degraded upon replication stress. Exponentially growing HeLa cells were first treated with either DMSO or 10 μM ATR inhibitor (VE-821) for 2 h and then exposed to 1 μM CPT for 1 h, washed with PBS and allowed to recover. Cells were harvested at indicated times and the total cell extract was analyzed for total WRN using anti-WRN polyclonal antibodies by western blotting. Ku80 was used as a loading control. **C.** The S1141A mutant WRN is stable in response to replication stress. Exponentially growing WS cells stably expressing Flag-EGFP tagged WT or Flag-EGFP tagged S1141A-mutant WRN were exposed to 1 μM CPT for 1 h, washed with 1x PBS and allowed to recover. Cells were harvested at indicated times and the total cell extract was probed with anti-Flag (monoclonal) antibodies by western blotting. Ku80 was used as a loading control. **D.** Phosphorylation of WRN at S1141 destabilizes WRN after replication stress. Exponentially growing WS cells stably expressing WT or S1141A-mutant WRN were co-treated with 30 μg/ml cycloheximide and 1 μM CPT for 1 h. Subsequently, cells were washed with 1x PBS, and maintained in growth medium containing cycloheximide. Cells were harvested at indicated times, and the total cell extract was probed with anti-Flag (monoclonal) antibodies. Ku80 was used as a loading control. **E.** WRN degradation is mediated by proteasomal pathway. Exponentially growing WS cells stably expressing WT or S1141A-mutant WRN were co-treated with 25 μM MG132 and 1 μM CPT for 1 h, washed with 1x PBS, and maintained in growth medium containing MG132. Cells were harvested at indicated times, and the total cell extract was probed with anti-Flag (monoclonal) antibodies. Ku80 was used as a loading control.

What makes phosphorylated WRN reversibly interact with replication-associated DSBs? Post-translational modifications change properties of a protein, either by affecting protein-protein interactions or facilitating additional modifications. Therefore, we investigated whether ATR-mediated WRN phosphorylation facilitates WRN ubiquitination after replication stress. For this purpose, we transiently transfected WS+WT and WS+S1141A WRN cells with HA-tagged ubiquitin and then treated cells with MG132 and CPT. WRN was then immunoprecipitated with anti-Flag antibodies and we examined the ubiquitinated WRN using anti-HA antibodies. Interestingly, we found that WT WRN was clearly ubiquitinated in response to CPT-treatment. In contrast, the S1141A mutation visibly attenuated replication stress-induced WRN ubiquitination (Figure 5A). Thus, ATR-mediated WRN phosphorylation induced by replication stress facilitates its ubiquitination.

Phosphorylation-dependent ubiquitination is the major protein degradation pathway in mammalian cells [45]. A previous study showed that ATM/NBS1-dependent WRN phosphorylation facilitates degradation of WRN following replication stress [17]. Therefore, we investigated the stability of WRN after replication stress. As shown in Figure 5B (left panel), the level of endogenous WRN was reduced gradually as a function of time in CPT-treated HeLa cells. In contrast, the level of endogenous WRN was stable in HeLa cells treated with CPT+ATR inhibitor (Figure 5B, right panel). We then evaluated the stability of the S1141A- S1141D-mutant WRN and WT WRN expressed in WS cells. The level of S1141A WRN increased gradually upon CPT exposure, whereas, the level of WT WRN was unchanged in response to replication stress (Figure 5C). Interestingly, the level of S1141D WRN decreased rapidly upon CPT exposure (Figure 5C). These results imply that the level of new WT WRN synthesis balances the degradation of WT WRN, whereas new S1141A-mutant WRN synthesis further increased the levels of stable S1141A WRN.

To validate the contribution of new protein synthesis to the cellular levels of WRN in response to replication stress, we inhibited new protein synthesis using cycloheximide and analyzed both WT and S1141A WRN. Levels of WT WRN were dramatically reduced in cells treated with both cycloheximide and CPT, but levels of S1141A WRN were only slightly reduced in cycloheximide and CPT-treated cells compared to those treated only with CPT (Figure 5D). Thus, WT WRN was degraded more rapidly than the S1141A WRN, suggesting that the S1141A-mutant WRN is more stable under replication stress conditions. To determine whether phosphorylated WRN is degraded through the ubiquitin-dependent proteasomal degradation pathway, we inhibited the 26S proteasome pathway with MG132. As shown in Figure 5E, pre-treatment of HeLa cells with MG132 followed by CPT treatment abolished WRN degradation, suggesting that WRN is degraded in a ubiquitin-dependent manner.

### ATR-mediated WRN phosphorylation at S1141 prevents chromosome instability

**Figure 6:**
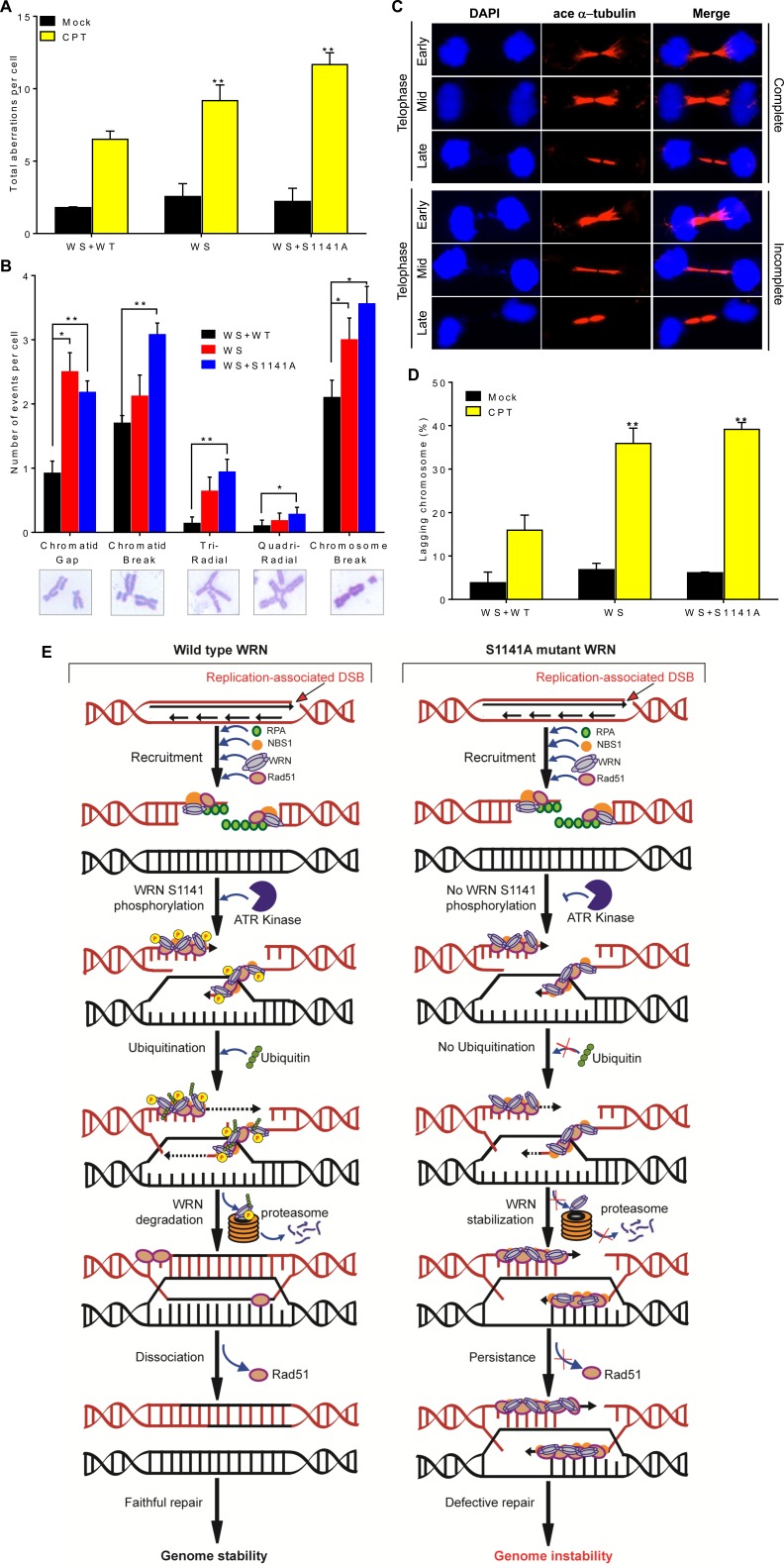
ATR-mediated WRN phosphorylation functions in genome stability maintenance **A.**-**B.** ATR-mediated WRN phosphorylation prevents chromosome instability. Exponentially growing WS, WS+WT, and WS+S1141A cells were treated with CPT for 1 h. After 12 h, chromosome preparations were made by accumulating mitotic cells in the presence of 0.1 mg/ml colcemid for 5 h. For each cell type, more than 50 metaphase spreads were counted. **A.** Total aberrations for each cell type. **B.** The number of types of aberrations per cell. Data was obtained by averaging results from three independent experiments. Error bars represent STDEV. * indicates P<0.005 and ** indicate *P* < 0.0005. **C.**-**D.** ATR-mediated WRN phosphorylation facilitates anaphase chromosome separation. **C.** Representative cell images show complete (top) and incomplete (bottom) chromosome separation in early-, mid- and late-telophases. **D.** Graph shows the percentages of cells with mis-separated chromosomes in WS, WS+WT, and WS+S1141A cells. Cells were treated with 1 μM CPT for 1 h. After 72 h, cells were fixed with 4% paraformaldehyde and incubated with acetylated anti-α−tubulin antibodies for indirect immunostaining. The average number of mis-segregated chromosome in > 100 acetylated α−tubulin-positive telophase junctions from three or four independent experiments was calculated. ace α−tubulin-acetylated α−tubulin; Error bars represent STDEV. ** indicate *P* < 0.001. **E.** Model depicting the mechanism of ATR-mediated WRN phosphorylation at S1141 in genome stability maintenance in response to replication-associated DSBs.

To investigate the involvement of WRN phosphorylation at S1141 in genome stability maintenance, we evaluated gross chromosomal aberrations in metaphase cells derived from WS, WS+WT, and WS+S1141A cells exposed to CPT. Classical chromosome analysis of metaphase spreads revealed that CPT-treated WS and WS+S1141A cells had significantly (*p* = 0.0008 and *p* = 0.0001, respectively) elevated levels of chromosomal aberrations per mitotic cells relative to WS+WT cells (Figure 6A). The average number of aberrations per cell was 10.2+0.5 and 11.7+0.6 for CPT-treated WS and WS+S1141A cells, respectively, and CPT-treated WS+WT cells had an average of ~6 per cell. More aberrations, including gaps, breaks, tri-radials, and chromosome breaks, were observed in WS and WS+S1141A cells than in WS+WT cells exposed to CPT (Figure 6B). Thus, ATR-dependent WRN phosphorylation at S1141 is important for preventing chromosome breaks in response to replication stress.

Presence of persistent γH2AX and RPA2 foci in WS and WS+S1141A cells in combination with other available data [46, 47] suggests that the resolution of recombination intermediates arising after RAD51-dependent strand invasion may be defective in WS and WS+S1141 cells. One consequence of this defect may be failure to resolve recombination and/or replication intermediates during or shortly after the S phase, causing an inability to undergo sister-chromatid disjunction during mitosis. A hallmark of failed chromosome segregation is the appearance of anaphase bridges, which arise from incompletely segregated chromosomal DNA connecting the daughter nuclei [48]. Therefore, we quantified chromosomal bridges in anaphase cells in mock- and CPT-treated WS, WS+WT, and WS+S1141A cells using acetylated alpha tubulin as a cytokinesis marker [49, 50] and DAPI to stain DNA (Figures 6C). We observed a very low frequency of anaphase bridges in mock- and CPT-treated WS+WT cells. In contrast, the frequency of anaphases with DAPI linkages between chromosomes was significantly higher in WS and WS+S1141A cells following CPT treatments as compared with WS+WT cells (*p* = 0.002 and 0.0005; Figure 6D). Our findings suggest the significance of ATR-mediated WRN phosphorylation in the faithful repair of replication -associated DSBs.

## DISCUSSION

Herein, we report identification and characterization of the biological role of a specific serine phosphorylation site in WRN. WRN is phosphorylated at S1141 in response to replication stress. We established that ATR is the kinase largely responsible for S1141 phosphorylation in response to collapsed replication forks. Biologically, ATR-mediated WRN phosphorylation functions in replication fork recovery, suppression of replication fork collapse, new origin firing, and replication-associated DSB repair. Mechanistically, WRN phosphorylation at S1141 causes reversible interaction of WRN with the collapsed replication forks and that facilitates faithful DNA replication and genome stability maintenance upon collapsed replication forks.

One *in vitro* study using anti-pS/TQ antibodies and analyzing random SQ/TQ mutations in WRN reported that S991, T1152 and S1256 residues are substrates of ATR kinase [27]. The same study also showed that S1054, S1141 and S1292 residues are substrates of ATM kinase [27]. Investigators did not make individual ATM and ATR site-specific point mutations in WRN to identify the biological functions of specific phosphorylation sites. However, by mutating multiple amino acid residues simultaneously and extrapolating results from multi-site mutants (2x, 3x, 4x and 6x) it was reported that phosphorylation by ATR regulates WRN sub-nuclear re-localization and interaction with RPA, preventing DSB formation at stalled forks. From other multi-site mutants the authors deduced that ATM phosphorylation promotes HR repair of collapsed forks by influencing the ability of Rad51 to form nuclear foci following exposure of cells to hydroxyl urea (HU). While these results are largely in agreement with our findings, a major difference is that S1141 is found to be phosphorylated by ATR, while the previous report ascribes phosphorylation to ATM. This discrepancy could be due to differences in the experimental approaches used, redundancy for ATR activity on WRN, or the influence of multiple mutations in WRN [27]. We used mass spectrometry to unambiguously identify serine at position 1141 as one of the WRN phosphorylation sites. We raised phospho-specific antibodies recognizing the phosphorylated S1141 and used this antibody to identify ATR as the kinase primarily responsible for WRN phosphorylation at S1141. We established that WRN phosphorylation at S1141 requires active DNA replication, and found that phosphorylation of WRN at S1141 is not essential for the recruitment of WRN, RPA2 and Rad51 to the sites of perturbed DNA replication. However, S1141 phosphorylation *in vivo* proved to be important for the dissociation of Rad51 and WRN from the damaged DNA ends. The ATR-mediated WRN phosphorylation led to WRN ubiquitination. Subsequent proteasome-mediated degradation was important for the suppression of chromosome instability in response to collapsed replication forks. Taken together our results show that WRN is one of the substrates of ATR kinase and that WRN phosphorylation by ATR is critical for the suppression of genome instability.

An unexpected finding of our study is that nascent DNA strands in CPT-treated cells expressing the mutant, non-phosphorylatable S1141A WRN were stabilized. Though most measured cellular phenotypes in WS+S1141A cells were similar to that of WRN-deficient cells, the nascent DNA strand stabilization function was unique to the S1141A-mutant WRN. In our previous study we reported that WRN-mediated stabilization of Rad51 at nascent DNA strands prevents MRE11-mediated degradation of newly replicated genome in response to replication-associated DSBs [11]. In this study we noticed that, unlike in cells lacking WRN, Rad51 nucleofilaments persisted in WS+S1141A WRN cells. Stable association of Rad51 at replication-associated DSBs could be the reason for the lack of nascent DNA stand degradation in WS+S1141A cells. Thus, these results further confirm our previous findings that WRN-mediated stabilization of Rad51 plays a role in the protection of newly replicated DNA.

Phosphorylation at S1141 may regulate WRN function by inducing conformational changes in the protein. Several other studies have shown that post-translational modification induces conformational changes in proteins involved in DNA metabolism that in turn alter its function [51-53]. Phosphorylation induces conformational changes by preventing the ability of certain proteins to interact with damaged DNA. In FRAP experiments, S1141A WRN, but not WT WRN, interacted with replication-associated DSBs in an irreversible manner. The reversible interaction of WT WRN with DNA at replication-associated DSBs may be required for timely and productive replication-associated DSB repair. Lack of dissociation of S1141A WRN might block access to other DNA repair enzymes, such as Mus81 [54], leading to incomplete resolution of recombination intermediates; however, further experiments are required to support this notion. Alternatively, it is also possible that phosphorylation might allow WRN to undergo additional modifications that may modulate its interaction with its binding partners.

Our data clearly indicate that S1141 phosphorylation leads to ubiquitination of WRN. Ubiquitination may affect protein-protein interactions [55]. Evidence indicates that WRN is recruited to the sites of replication-associated DSB by NBS1 [11]. Therefore, we speculate that phosphorylation-dependent ubiquitination of WRN may regulates its interaction with NBS1. Also, the absence of S1141 phosphorylation leads to a tight interaction between WRN and NBS1. This could be one of the reasons why the S1141A-mutant WRN persists at the sites of replication-associated DSBs. However, further experiments are need to verify these speculations. Ubiquitination also targets proteins for proteasome-mediated degradation [45]. In support of this function we found that the S1141 phosphorylated WRN was degraded more rapidly than the S1141A-mutant WRN. It was previously shown that ATM/NBS1-dependent WRN phosphorylation facilitates degradation of WRN in response to replication stress [17]. Our data indicate that ATR-mediated WRN phosphorylation results in WRN ubiquitination, influencing its interaction with its binding partners and its stability.

We found that phosphorylation of WRN at S1141 is essential for the proper segregation of chromosomes during anaphase, yet the impact of ATR-mediated phosphorylation of WRN in anaphase chromosome separation is unclear. Proper sister chromatid separation by a bipolar mitotic spindle is required to generate two identical daughter cells. During anaphase, all physical connections between sister chromatids must be resolved and failure to resolve linkages may lead to incomplete anaphase chromosome separation [56-58]. Our data clearly show that the recruitment of RPA2, RAD51, and WRN to the sites of replication-associated DSBs was not affected by the phosphorylation status of WRN; however, due to high DNA binding affinity of the S1141A-mutant WRN, WRN and Rad51 persist at CPT-induced DSBs, possibly affecting subsequent steps of HR-mediated repair. Therefore, the presence of unresolved DNA lesions in WS+S1141A cells likely leads to incomplete anaphase chromosome separation. In support of our findings, a number of cell lines deficient in distinct steps of the HR pathway exhibit elevated levels of chromosome mis-segregation. For example, cells defective in BRCA2 [59, 60], BLM [48], XRCC2, XRCC3 [61] and FANCD2 [50], but not cells defective in the non-homologous end joining pathway [57], exhibit incomplete anaphase chromosome separation.

Mutations in *WRN* gene lead to WS. Individuals with this syndrome are at risk for developing cancer. What is the link between a defect in resolving replication blockage and genome instability? The presence of a large number of chromosomal aberrations in CPT-treated WRN and S1141 phosphorylation defective cells suggests that faithful processing of replication forks is critical for the prevention of chromosomal instability. Though exposure of WS and WS+S1141A cells to CPT compromises cell survival (Figure 2B), some cells with chromosomal aberrations enter mitosis. Every subsequent round of replication is expected to increase the overall mutation level in surviving cells. Additionally, a single-nucleotide polymorphism at 1141 altering serine to leucine has been reported in patients with acute myeloid leukemia [62]. Thus, the biological significance of defective replication fork processes is high, because replication of a damaged genome can provide the opportunity for genomic rearrangements and can increase genomic instability leading to genetic changes required for progression from an initiated cell to a malignant cell.

WS is a hereditary disease featuring a broad spectrum of premature aging pathologies and predisposition to many different cancer types [63]. Hypomorphic mutations in ATR have been linked to Seckel syndrome in humans, a disease characterized by severe growth retardation, microcephaly, and facial and osteoskeletal abnormalities [64]. Similar to cells derived from WS patients, ATR-Seckel fibroblasts grow slowly, and exhibit slow cycling time, increased chromosomal instability and increased replication fork stalling [65]. These studies indicated that ATR plays an important role in maintaining genome integrity during DNA synthesis, by stabilizing stalled DNA replication forks and preventing their collapse into DSBs [66-68]. As a central upstream regulator of cellular responses to replication stress and DNA damage, ATR phosphorylates many DNA damage response factors, including WRN, to maintain genome stability. The interaction between ATR and WRN in a common signaling pathway, the resemblance between WS and ATR-Seckel cells, and the potential involvement of aberrant DNA replication in both syndromes strongly suggest a functional relationship between WRN and ATR. In our current study, ablation of a single ATR mediated phosphorylation site in WRN comprises many of the cellular phenotypes of WS, including a reduced rate of replication fork progression, unrepaired DSBs and higher levels of chromosome aberrations after CPT exposure, and hypersensitivity to CPT. WRN is a multifunctional protein, our current data and other reports suggest that WRN is downstream of the ATR signaling cascade. Therefore, it will be interesting to pin-point whether ATR mediated WRN phosphorylation is required for all the functions of WRN or necessary for only a sub-set of WRN functions.

We propose a model for ATR-mediated WRN phosphorylation in the suppression of genomic instability in response to replication-associated DSBs (Figure 6E). NBS1 recruits WRN to the sites of replication-associated DSB [11] and WRN is phosphorylated by ATR at S1141 after replication stress. Upon phosphorylation by ATR, WRN is ubiquitinated modulating its interaction with replication-associated DSBs (probably by affecting the NBS1-WRN interaction). This in turn facilitates replication fork processing and HR-mediated replication-associated DSB repair by granting access to factors (probably Mus81/Eme1) involved in replication and repair. Finally, post-translationally modified WRN is targeted to the proteasome-dependent degradation pathway. Ultimately, all these processes lead to genome stability maintenance upon replication stress. It is worth mentioning that a single-nucleotide polymorphism at 1141 altering serine to leucine has been reported in patients with acute myeloid leukemia [62]. In the absence of ATR-dependent WRN phosphorylation, ubiquitination of WRN is attenuated, leading to the stable interaction of WRN with replication-associated DSBs likely preventing binding of other factors involved in replication and repair. As a consequence, cells cannot resolve recombination intermediates arising after RAD51-dependent strand invasion. These events eventually lead to anaphase bridge formation and chromosome instability.

We presented evidence that ATR-mediated WRN phosphorylation at S1141 is critical for DNA replication, repair, and recombination. Our findings on the defects in replication fork processes, replication-associated DSB repair, and chromosome stability are remarkable because a single amino acid change (S1141A) in WRN induces phenotypes which are indistinguishable from cells missing the entire WRN function. Furthermore, the similarity between WS and ATR-Seckel cells and the potential involvement of ATR-mediated WRN phosphorylation in suppressing cellular phenotypes of WS suggest that WRN functions as a signal transducer of the ATR-mediated signaling cascade in a subset of processes involved in replication-associated genome stability maintenance. ATR-mediated WRN phosphorylation at S1141 and subsequent ubiquitination of WRN are critical for faithful and timely DNA replication, repair, and recombination after replication stress.

## MATERIALS AND METHODS

### Cell lines

The simian virus 40 transformed control (AG07217A) and Werner Syndrome (AG11395) fibroblasts were obtained from the Coriell Institute for Medical Research. WS cells immortalized by expression of the human telomerase reverse transcriptase and WS cells complemented with wild-type WRN were described previously [11]. HT1080-1885 cells carrying a single copy of the HR substrate were described previously [43]. All cell lines were grown in standard tissue culture conditions in 5% CO_2_ and were maintained in Dulbecco's modified Eagle's medium supplemented with 10% fetal bovine serum, 2 mM glutamine, and 0.1 mM non-essential amino acids. To establish stable cell lines, WS-SV40 cells were infected with a retrovirus carrying WT, S1141A, or S1141D full-length WRN fused to Flag and EGFP and placed under puromycin selection (0.1 μg/ml). Stable clones were isolated, and WRN expression was evaluated by western blotting and live cell imaging. Retroviral packing and infection was carried out according to the manufacturer's instructions (Clontech). AMAXA Nucleofector (Solution T and program L005) was used for transient expression of Flag-tagged WRN (pBICEP, Sigma) and shRNA mediated inhibition of *WRN* expression in HT1080-1885 cells.

### Identification of WRN phosphorylation sites

WRN and Ku proteins were purified to near homogeneity from Sf9 insect cells infected with recombinant baculovirus carrying the respective human cDNAs [24]. DNA-PKcs was purified from either human placenta or cultured HeLa cells as reported previously [69]. Purified WRN was dephosphorylated using a mixture of λ-phosphatase and protein phosphatase 1 (New England Biolabs). Purified WRN was phosphorylated *in vitro* by DNA-PKcs in the presence of Ku70/80 and 50 μM ATP. The phosphorylated WRN band was excised, digested with trypsin, and analyzed by mass spectrometry as described previously [33].

### DNA manipulation and construction of the expression vectors

Standard molecular biology procedures were used to construct all plasmids. The serine to alanine and aspartic acid mutants of WRN were generated by site-directed mutagenesis using primers designed to create the mutation at the desired position by changing the TCA codon encoding a serine either to a GCA codon encoding alanine or to a GAC codon encoding aspartic acid. Retroviral vectors containing full-length WT, S1141A, or S1141D WRN were constructed by inserting PCR fragments containing the human WT, S1141A, and S1141D WRN into Xho I sites of the modified retroviral vector (pQXIC-EGFP). Mammalian expression vectors were constructed by inserting PCR fragments containing the human WT, S1141A, or S1141D WRN into Sal I sites of the mammalian expression vector (pBICEP, Sigma). All constructs were sequenced to confirm desired mutations using a number of primers sets covering the different regions of the WRN cDNA.

### Cell extracts and western blotting

Nuclear extracts (P10) were prepared as described previously [69]. Whole-cell extracts were prepared by suspending cell pellets in RIPA buffer on ice for 20 min followed by centrifugation to remove insoluble material. All buffers contained phenylmethanesulfonylfluoride (PMSF), aprotinin, leupeptin, pepstatin A, sodium fluoride (NaF), and sodium orthovanadate (Na_3_VO_4_) at 1 μg/ml. Approximately 20-100 μg of nuclear or whole-cell extracts were resolved by 6-10% SDS-PAGE, transferred onto nitrocellulose membrane, and reacted with different antibodies.

### Protein stability and ubiquitination assays

For protein stability assay, WS+WT and WS+S1141A cells were co-treated with 30 μg/ml cycloheximide (Sigma) and 1 μM CPT (Sigma). For WRN ubiquitination assay, WS+WT and WS+S1141A cells were transfected with HA-Ub plasmid using Lipofectamine 2000 (Invitrogen). After 36 h, cells were treated with 25 μM MG132 (Calibiochem) and 1 μm CPT for 1 h. Cells were washed with 1xPBS and maintained in growth medium containing MG132 for 16 hours. Cells were harvested and WRN was immunoprecipitated from the total cell extract using anti-Flag (Sigma) antibodies. Subsequently, the immune complex was separated onto a 6% SDS-PAGE and probed with anti-HA and anti-WRN antibodies.

### Antibodies

Phospho-S1141 (pS1141) polyclonal antibodies were prepared by immunizing New Zealand white rabbits with the phosphopeptide EKAYSSS[PO3]QPVISA conjugated to keyhole limpet hemocyanin (KLH). The phospho-specific antibodies were affinity-purified through a phospho-peptide-conjugated Sepharose CL-4B column. Eluted IgGs were then passed through the corresponding unphosphorylated peptide column to deplete any IgGs that were not specific for pS1141. An anti-WRN monoclonal antibody (mAb) was raised against the 940-1432 aa region of WRN and a polyclonal antibody (pAb) was raised against the 1-250 aa region of WRN [11]. Commercially available anti-γH2AX mouse monoclonal antibody (Upstate Biotechnology), anti-RPA2 mouse monoclonal antibody (Calbiochem), anti-Rad51 rabbit polyclonal antibody (Calbiochem), anti-BrdU mouse monoclonal antibody (BD, 347580), and anti-Flag (M2) monoclonal (Sigma) antibodies were used. Fluorescent conjugated secondary antibodies (Alexa-488, Alexa-555, Alexa-633) were purchased from Molecular Probes (Invitrogen).

### Indirect immunostaining

Approximately 2 × 10^5^ cells were plated in six-well plates containing cover glasses and incubated for 36 h. Cells were treated with 1 μM CPT for 1 h, washed twice with PBS, and then allowed to recover in CPT-free growth medium. Cells were fixed with 4% paraformaldehyde (PFA) for 20 min at room temperature at different post-CPT recovery times and subjected to indirect immunofluorescence as described previously [11]. For the localization of WRN, cells were washed three times in PBS and incubated in extraction buffer (50 mM HEPES, pH 7.4, 150 mM NaCl, 1 mM EDTA, 0.5% NP40) on ice for 10 min prior to fixation. For the localization of RPA2 and RAD51, cells were washed three times in PBS and incubated in extraction buffer (10 mM HEPES, pH 7.4, 300 mM sucrose, 100 mM NaCl, 3 mM MgCl_2_, 0.1% Triton X-100) on ice for 10 min prior to fixation. For immunostaining, cells were permeabilized in Triton X-100 (0.5% in PBS) on ice for 10 min, washed three times with PBS, incubated in blocking solution (5% goat serum in PBS) at room temperature for 60 min, and then incubated with primary antibodies (diluted in 5% goat serum) at room temperature for another 3 h. Subsequently, cells were washed with 1% BSA in PBS, incubated with appropriate secondary antibodies (1:800 in 2.5% goat serum, 1% BSA, and PBS) at room temperature for 1 h, washed five times with 1% BSA, and mounted with mounting medium containing DAPI (Vectashield).

### Microscope image acquisition and foci dissolution kinetics assay

Images were captured using an LSM 510 Meta laser scanning confocal microscope with a 63X1.4 NA Plan-Apochromat oil immersion objective. Images were taken at z-sections (15-20 sections) of 0.3-μm intervals using the 488-nm (Alexa 488), 543-nm (Alexa 555), 633-nm (Alexa 633), and 405-nm (DAPI) lasers. The tube current of the 488-nm argon laser was set at 6.1 A. The laser intensity was typically set to 3-5% transmission of the maximum intensity with the pinhole opened between 1 (for foci) and 2 (for nuclei) Airy units. For WRN, RPA2, RAD51, and γH2AX foci counting, the z-sections were assembled using the Imaris software and quantified as described previously [39]. Quantification of foci was conducted from images of 20-100 cells for each time point from two or three independent experiments.

### EdU labeling

Approximately 3 × 10^5^ WS cells stably expressing EGFP-WT and EGFP-S1141A WRN were plated in six-well plates containing cover glasses (22 × 22 mm) and incubated for 36 h. Cells were pulsed with 50 μM EdU for 90 min in growth medium before camptothecin (CPT) treatment. Cells were washed twice with PBS and exposed to 1 μM CPT. After 8 h, cells were washed twice in PBS and fixed with 4% PFA for 20 min at room temperature. Cell fixation and immunostaining were carried out as described in indirect immunostaining section. For EdU detection, we used the Click-It reaction kit (Invitrogen) prior to secondary antibody detection.

### Clonogenic cell survival

WS cells stably expressing EGFP, EGFP-WT, EGFP-S1141A, and EGFP-S1141D WRN were plated in triplicates (3 × 10^2^-2 × 10^3^ cells/25-cm^2^ flask) and incubated for 24 h. Cells were then treated with varying concentrations of CPT for 72 h. Cells were washed twice, trypsinized, counted, and replated in three 10-cm^2^ dishes. After a 12-day incubation, dishes were stained with crystal violet (0.5% crystal violet, 1% formaldehyde, 1X phosphate buffered saline), and survival was scored by counting colonies. Survival curves were generated from three independent experiments with colony numbers normalized to sham-treated controls.

### Homologous recombination assay

This assay was carried out as described previously [43]. Briefly, human HT1080-1885 cells carrying a I-SceI-inducible single copy of a homologous recombination substrate were transfected with either I-SceI expression vector pCMV (3 × NLS) alone or with *WRN* shRNA, Flag tagged WT WRN, or S1141A WRN using Amaxa Nucleofector (Solution T, Program L005). After 24 h, cells were replated in triplicate for puromycin selection at 120,000 cells/100-mm culture dish. Parallel platings for efficiency measurement were made in duplicate at 250 cells/100-mm dish. On days 2, 6, and 8 after transfection, medium in selection cultures was replaced with fresh medium containing 1 μg/ml puromycin. On day 13, cells were fixed and stained. Cultures for the plating efficiency assay were incubated at 37°C for 11 days without refeeding and then were fixed and stained. Frequency of HR was scored by counting puromycin-resistant colonies. HT1080-1885 is a clonal isolate of HT1080 cells stably carrying the reporter and produces spontaneous puromycin-resistant colonies at a frequency of ~1 × 10^7^/viable cells without I-SceI expression.

### Metaphase spreads

At 12 h after CPT treatment, chromosome preparations were made by accumulating metaphases in the presence of 0.1 μg/mL colcemid (Irvine Scientific) for 5 h. Cells were trypsinized and then washed once with PBS and incubated in 10 mL of hypotonic solution (0.075 M KCl) at 37°C for 15 min. Cells were pre-fixed with 1/10 volume of ice-cold methanol:acetic acid (3:1 ratio) in hypotonic solution and then centrifuged at 800 × g for 5 min at 4°C. Subsequently, cells were fixed with methanol:acetic acid (3:1 ratio) on ice for 30 min and then kept at −20°C until use. Cells were dropped onto pre-cleaned cover glasses, stained with Giemsa stain (5%; KaryoMAX, GIBCO) for 5 min at room temperature, and then washed with dH_2_O. Images were taken using an Olympus microscope (100X objective) equipped with an Image Spot camera (Spot Imaging Solutions).

### Fluorescence recovery after photobleaching (FRAP)

FRAP analysis was performed on an LSM 510 Meta confocal microscope as described previously [39]. Briefly, cells expressing EGFP-WT, S1141A, or EGFP-S1141D WRN were treated with CPT (1 μM) for 1 h. CPT-containing medium was removed and cells were washed three times with growth medium and were allowed to recover at 37°C for 8-24 h to allow maximum accumulation of EGFP-WRN at the DSB sites. Subsequently, 1 or 2 EGFP-WRN foci were photo-bleached with a pulse of a 488-nm argon laser. Time-lapse imaging before and after photobleaching and fluorescence quantification were performed as described previously [39]. All measurements were corrected for nonspecific monitor bleaching.

### DNA fiber assay

The DNA fiber assay was carried out as described previously [11, 50]. Briefly, ~2.5 × 10^5^ and 1 × 10^6^ cells were platted in a six-well plate (labeled) and 10-cm dishes (unlabeled), respectively, and incubated for 24 h. Cells in six-well plates were first labeled with IdU (150 μM) for 30 min, washed four times with warm PBS, exposed to CPT (1 μM) for 1 h, washed three times with growth medium, and then either labeled with CldU (150 μM) for 30 min or allowed to recover for an additional 5 h. After three washes with warm PBS, both labeled and unlabeled cells were trypsinized and counted. Cell numbers were adjusted to a final concentration of 10^6^/ml and mixed at 1:20 ratio (labeled to unlabeled). Cells were lysed on a clean glass slide in 20 μl of lysis buffer (0.5% SDS, 50 mM EDTA and 200 mM Tris-HCl pH 7.4) for 8 min and slides were tilted slightly (~15° angle) to help DNA spread slowly. After air drying the samples, DNA was fixed with methanol:acetic acid (3:1) at room temperature for 8-10 min. Slides were air-dried and kept in 70% ethanol at 4°C until use. For immunostaining, slides were first washed three times with PBS at room temperature. To denature DNA, slides were incubated in 2.5 N HCl in a glass jar at 37°C for 50 min and then neutralized by washing four times with PBS. Following neutralization, slides were incubated in 5% goat serum in PBS for 2 h at room temperature. Subsequently, mouse and rat anti-BrdU antibodies were diluted in 5% goat serum, 0.1% Triton X100, and PBS and incubated at 37°C for 1 h in a humidified chamber, washed three times in PBS containing 0.1% TritionX100 and incubated with anti-rat Alexa 488 and anti-mouse Alexa 555 in 5% goat serum and 0.1% Triton X-100 for 1 h. After washing three times with PBS containing 0.1% Triton X100, slides were mounted in mounting medium without DAPI (Vectashield). Images were acquired using a fluorescent microscope (Zeiss), and the DNA fiber lengths were measured using Axiovision Software.

### Statistical analysis

Data are expressed as means ± SEM or STDEV of at least two independent experiments. The Student's *t*-test was performed to calculate the level of significance and a value of *p* < 0.05 was considered statistically significant. GraphPad Prism (version 6.0) was used to calculate DNA-fiber-length distribution and for making the graphs.

## SUPPLEMENTARY MATERIAL FIGURES


