# Supplementary material for: Replication stress induced site-specific phosphorylation targets WRN to the ubiquitin-proteasome pathway
Source: Oncotarget. 2015 Dec 18;7(1):46–65. doi: 10.18632/oncotarget.6659 (PMC4807982; doi:10.18632/oncotarget.6659)
Supplement: Supplementary file 1 [file oncotarget-07-0046-s001.pdf]

Replication stress induced site-specific phosphorylation targets WRN to the ubiquitin-proteasome pathway

Supplemental Material

Experimental procedures

BrdU Labeling and Flow Cytometry: Cells were pulsed with 30 μM BrdU for 30 min in growth medium before CPT treatment. Cells were washed twice with PBS and exposed to CPT (25nM) for 1 hour. At different time points after CPT treatment, cells were washed twice in PBS and fixed with ice-cold 70% ethanol at –20°C overnight. For BrdU flow cytometry, cells were first washed twice with cold PBS and then treated with 100 μg/mL RNase/5% Tween 20 in PBS at 37°C for 30 min. To denature DNA, cells were resuspended in 4 M HCl (1 mL per 10^6 cells) containing 0.5% Tween 20, incubated at 37°C for 30 min, and then neutralized by washing twice with PBS. Following neutralization, cells were incubated in 100 μL of PBS containing 0.1% Tween 20, 1% BSA, and anti-BrdU antibody conjugated with FITC (1:100; Molecular Probes) at room temperature for 2 h. After washing with 1% BSA, cells were stained with 10 μg/mL propidium iodide (PI; Sigma) at room temperature for 30 min and subjected to flow cytometry (FACScan, Becton Dickinson).

Neutral comet assay: Cells with various WRN mutations (WT, WS, 1141A and 1141D) were treated with 1μM CPT. After 1h of treatment, CPT was removed by washing the cells in warm PBS thrice and cells were allowed to recover in normal growth media. At different times points after CPT treatment, cells were trypsinized, embedded in low melting agarose and subsequently lysed and processed according to the manufacturer's protocol (Trevigen, Catalog # 4250-050-K). If DNA double strand breaks have occurred, the propidium iodide-stained nuclei will display a comet-like tail emanating from the cell nucleus, corresponding to fragmented DNA. Images were taken using a Zeiss fluorescence microscope. Comet tail lengths were quantified using Image J software. We eliminated apoptotic cells (smaller comet head and extremely larger comet tail) from the analysis.

Supplemental Results

WRN phosphorylation at S1141 is important for S-phase progression: WS cells display a prolonged S-phase and impaired replication fork progression (1). To determine whether ATR mediated WRN phosphorylation influences S-phase progression, we pulse-labeled WS, WS+WT and WS+S1141A cells with BrdU and measured the progression of S-phase cells by fluorescence-activated cell sorting **(Fig. S4)**. Mock-treated WS, WS+WT and WS+S1141A cells moved through S-phase and reached G2/M-phase within 8 h, progressing from the G2/M to the G1 phase within 16 h **(Figs. S4A-C).** In response to CPT treatment, WS and WS+S1141A cells, but not the WS+WT cells, exhibited a marked delay in progression through the S phase. The majority of WS and WS+S1141A cells were arrested in the S-phase for more than 16 hrs and only a small fraction of S-phase cells progressed to the G2 phase by 24 h post CPT-treatment **(Figs. S4D-F).** In contrast, WS+WT cells progressed through the S-phase very quickly, while the majority of S-phase cells progressed to G2 by 8 h **(Figs. S4D-F).** These results clearly demonstrate that S1141 phosphorylation plays a critical role in the progression of S-phase cells in response to collapsed replication forks.

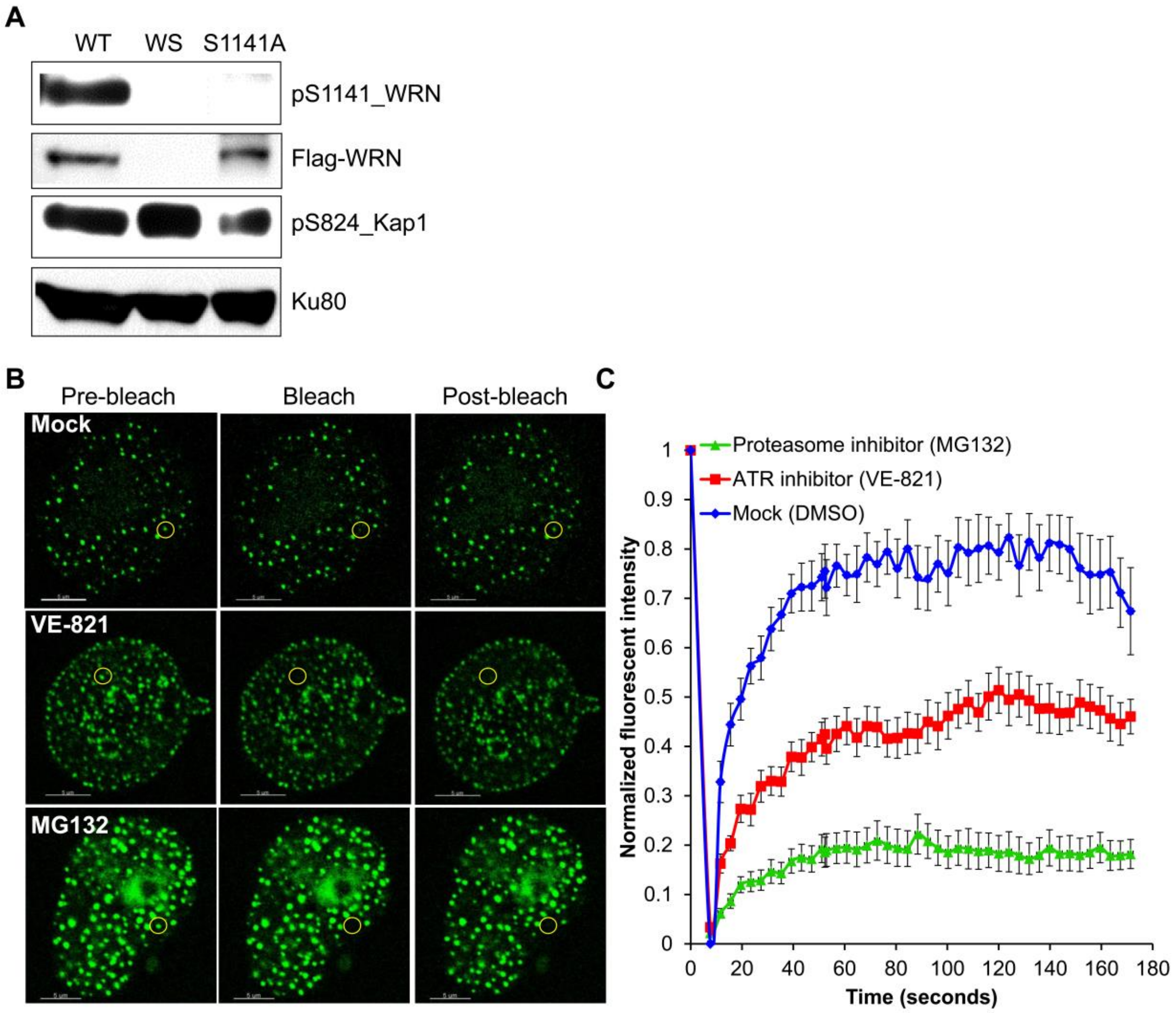

Figure S1

A. S1141A WRN is not phosphorylated in response to CPT treatment: WS cells stably expressing WT and the S1141A mutant were CPT-treated (1 μM) for 1 h. Cells were harvested 1 h after treatment and analyzed for phosphorylation of pS1141-WRN and KAP1 by western blotting.

B-C. ATR-mediated WRN phosphorylation modulates the association of WRN with DSBs. **(C)** Representative confocal images show EGFP-WT WRN foci before and after photobleaching (circled are representative foci) in cells treated with ATR (top, 10 μM VE-821) and proteasome (bottom, 25 μM MG132) inhibitors. **(D)** FRAP curves for EGFP-WT WRN foci in cells treated with ATR and proteasome inhibitor are shown. WS cells stably expressing EGFP-tagged WT were treated with 1 μM

CPT for 1 h. Cells were allowed to recover for 24 h, and one or two randomly selected WRN foci were photobleached. The recovery of fluorescence signal was captured using a live cell confocal microscope. Cells were treated with 10 μM VE-821 four hours before CPT treatment to inhibit ATR; cells were treated with 25 μM MG132 8 hr after CPT-treatment to inhibit proteasome mediated degradation of WRN. In every image, average fluorescent intensities of the photobleached EGFP-WRN foci were measured as a function of time and then divided by the average fluorescent intensity measured elsewhere in the cell as a function of time. Normalized FRAP curves for each cell were obtained by dividing EGFP-WRN fluorescent intensities in each spot after photobleaching by the pre-bleach intensity; the pre-bleach intensity was set to 1. Each data point depicted in the graph is the average of 20 independent, normalized measurements. The error bars represent SEM.

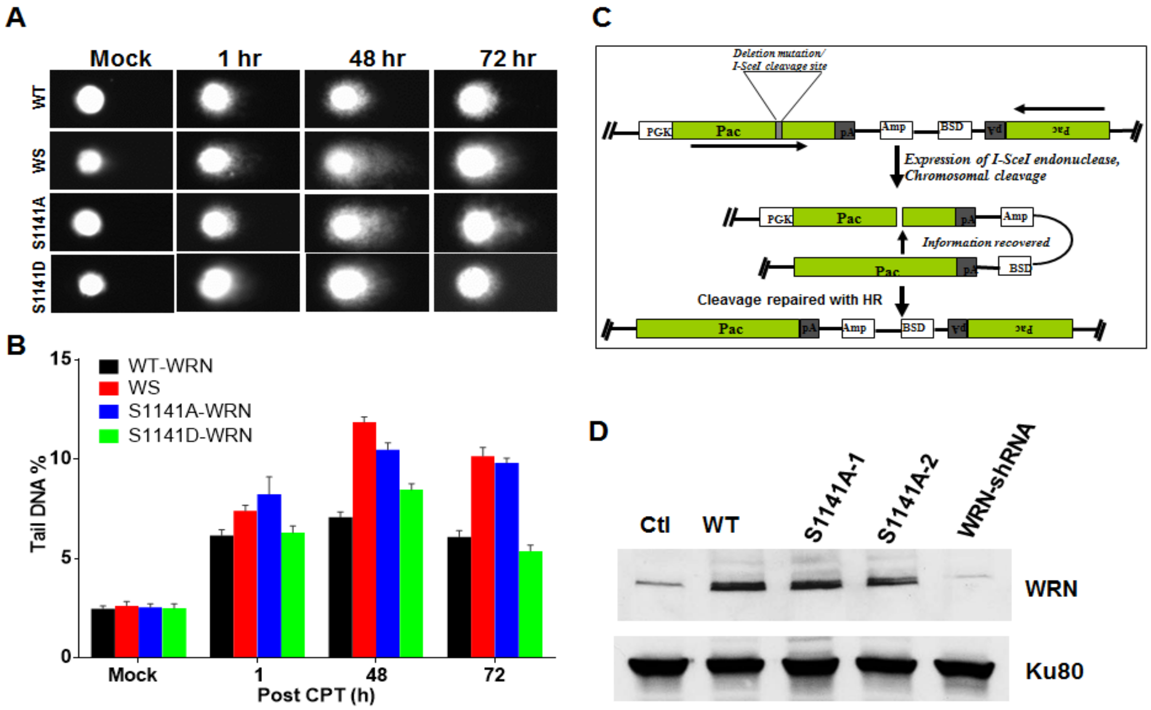

Figure S2

A-B. Replication-associated DSBs are difficult to repair in WS cells stably expressing S1141A WRN. A. Representative images show neutral comet assay. **B.** Average comet tail movement in WS, WS+WT and WS+S1141A WRN cells is shown. Exponentially growing WS and WS cells stably expressing WT, S1141A and S1141D WRN were exposed to 1 μM CPT for 1 hr and cells were harvested at indicated times after the CPT-treatment. Subsequently, cells were subjected to neutral comet assay. Each data point in the graph is the average of 20 cells. Error bars represent SEM.

C. Graphical sketch showing homologous recombination substrate containing mouse *phosphoglycerate kinase* (*PGK*) enhancer/promoter. Pac, the *puromycin acetyltransferase* gene, confers puromycin resistance and deletion or mutation of I-SceI cleavage site inactivates the *Pac* gene. A is the SV40 polyadenylation region; Amp is the bacterial origin of replication and ampicillin resistance gene (needed for propagation of the vector as a plasmid); and BSD confers blasticidin resistance gene. *BSD* is under the control of the cytomegalovirus promoter/enhancer and

bovine growth hormone poly(A) signal. Diagonal parallel lines indicate flanking chromosomal sequences.

D. Western blots show expression of WRN in HT1080-1885 cells harboring a single copy of the I-SceI-inducible HR substrate. HT1080-1885 cells harboring a single copy of the I-SceI-inducible HR substrate were transfected with either I-SceI expression vector pCMV (3 × NLS) alone or with *WRN* shRNA, Flag tagged WT WRN, or S1141A WRN using Amaxa Nucleofector (Solution T, Program L005). Two days after transfection, cells were harvested and subjected to western blotting analysis with WRN and Ku80 antibodies.

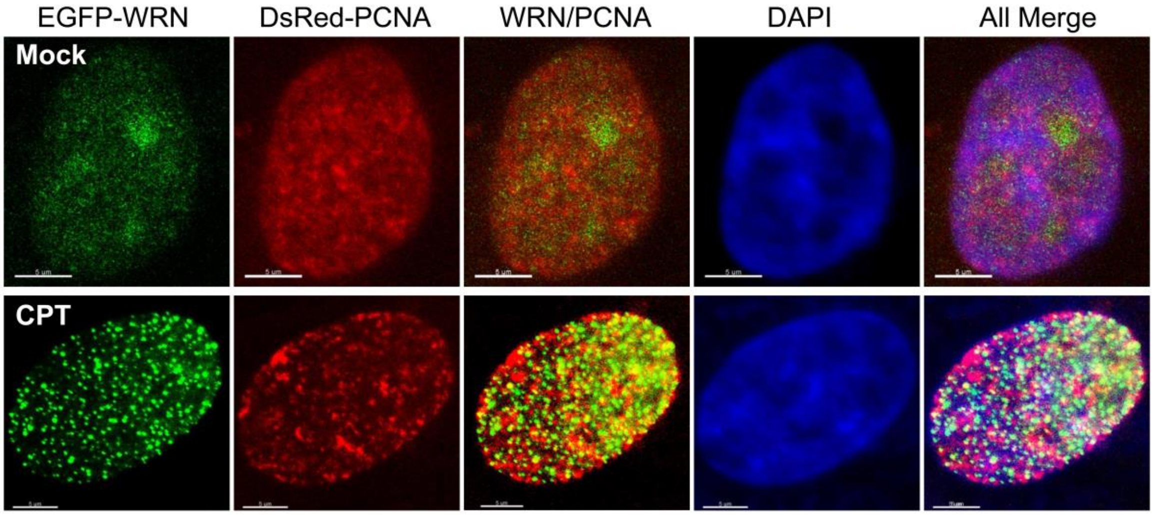

Figure S3

WRN is co-localized with PCNA in response to CPT-treatment: WS cells stably expressing EGFP-WT WRN were transiently transfected with DsRed-PCNA and then treated with 1 μM CPT for 1 hr. After 8 h, cells were fixed with 4% paraformaldehyde and imaged using a confocal microscope. Representative three-dimensional deconvoluted confocal images are shown. Scale bars are 5 μm

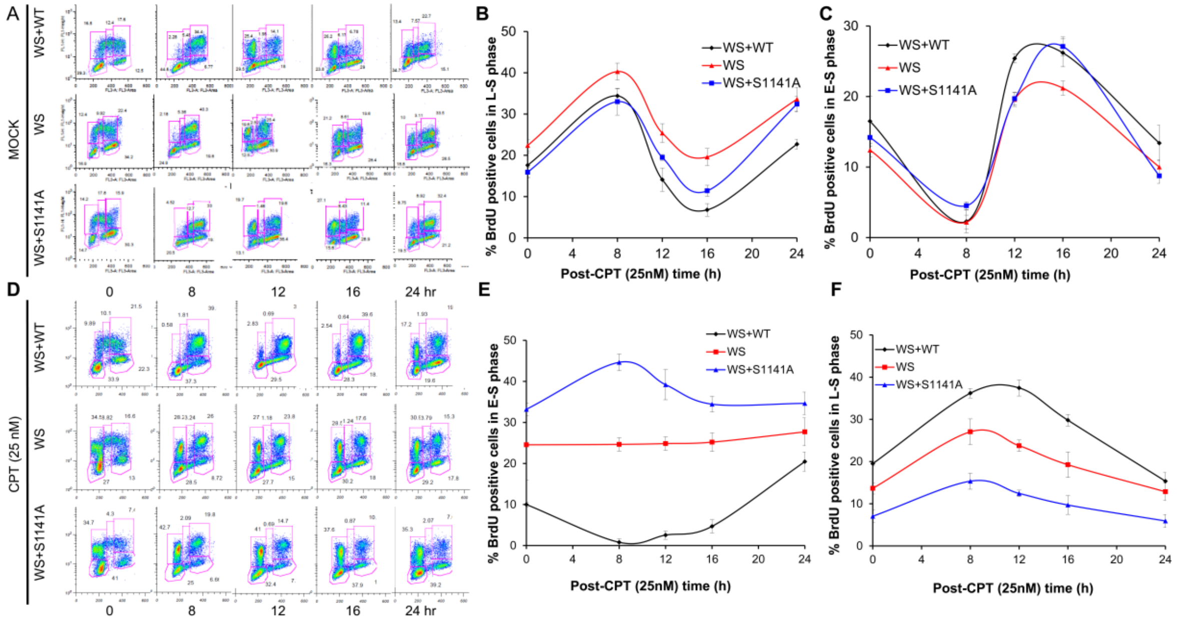

Figure S4

A-F. ATR-mediated WRN phosphorylation is required for S-phase progression in response to collapsed replication forks. Exponentially growing WS and WS cells stably expressing WT and S1141A WRN were pulse-labeled with BrdU for 30 min and then exposed to CPT (25 nM) for 1 h and cells were harvested at indicated times after the CPT-treatment. Subsequently, cells were immunostained with FITC-conjugated anti-BrdU antibody and then subjected to flow cytometry. Each data point in the graph is the average of three independent experiments. E-S: early S-phase; L-S: late S-phase. Error bars represent STDEV.

Supplemental References

1. Sidorova, J.M., Kehrli, K., Mao, F. and Monnat, R., Jr. (2013) Distinct functions of human RECQ helicases WRN and BLM in replication fork recovery and progression after hydroxyurea-induced stalling. *DNA Repair (Amst)*, **12**, 128-139.
